# The Associations Between Access to Recreational Facilities and Adherence to the American Heart Association's Physical Activity Guidelines in US Adults

**DOI:** 10.3389/fpubh.2021.660624

**Published:** 2021-11-24

**Authors:** Larissa Andrade, Ryan Geffin, Mark Maguire, Pura Rodriguez, Grettel Castro, Ahmad Alkhatib, Noël C. Barengo

**Affiliations:** ^1^Department of Translational Medicine, Herbert Wertheim College of Medicine, Florida International University, Miami, FL, United States; ^2^School of Health and Life Sciences, Teesside University, Middlesbrough, United Kingdom; ^3^Department of Public Health, Faculty of Medicine, University of Helsinki, Helsinki, Finland; ^4^Department of Health Policy and Management, Robert Stempel College of Public Health and Social Work, Florida International University, Miami, FL, United States

**Keywords:** open space access, recreational facilities, built environment, sedentarism, compliance

## Abstract

Physical activity decreases the risk of long-term health consequences including cardiac diseases. According to the American Health Association (AHA), adults should perform at least 75 min of vigorous physical activity (PA) or 150 min of moderate PA per week to impact long-term health. Results of previous studies are varied and have yet to integrate perceived access to facilities with AHA PA guidelines. We investigated whether access to free or low-cost recreational facilities was associated with meeting the AHA PA guidelines.

**Methodology:** This cross-sectional study utilized data extracted from the Family Life, Activity, Sun, Health, and Eating (FLASHE) database collected in 2017 (*n* = 1,750). The main exposure variable was access to free or low-cost recreational facilities. The main outcome variable was meeting the AHA guidelines of 150 min moderate PA or 75 min vigorous PA per week. Covariates included age, sex, level of education, overall health, BMI, ethnicity, hours of work per week, income, and time living at current address. Unadjusted and adjusted logistic regression analysis were used to calculate measures of odds ratio (OR) and corresponding 95% confidence interval (CI).

**Results:** Of the 1,750 included participants, 61.7% (*n* = 1,079) reported to have access to recreational facilities. Of those with access to facilities, 69.9% met AHA PA guidelines while 30.4% did not. After adjusting for covariates, participants who reported access to recreational facilities were 42% more likely to meet AHA PA guidelines compared with participants who did not (adjusted OR 1.42; 95% CI 1.14–1.76). Secondary results suggest that healthier individuals were more likely to have met AHA PA guidelines.

**Conclusions:** Having access to free or low-cost recreational facilities such as parks, walking trails, bike paths and courts was associated with meeting the AHA PA guidelines. Increasing prevalence and awareness of neighborhood recreational facilities could assist in access to these facilities and increase the ability of individuals to meet AHA PA guidelines. Future research should determine which types of recreational facilities impact physical activity strongest and discover methods of increasing their awareness.

## Introduction

Physical activity (PA) is known to decrease the risk of developing long term chronic diseases such as cardiovascular disease. Inadequate PA has long been associated with disease-related mortality and increased healthcare costs. Recent estimates in the US reported about 10% of pre-mature deaths were associated with inadequate PA, and $117 billion in annual healthcare costs (1). The current recommendation of the American Heart Association (AHA) for adults is to perform at least 150 min of moderate PA or 75 min of vigorous PA per week to have an impact on long term health (2). However, the U.S. Department of Health and Human Services (HHS) estimated that more than 80% of adults do not currently meet such guidelines (3). In such, it is essential to understand the factors associated with meeting current US guidelines among US populations.

One of the commonly accepted factors for increasing PA participation among populations is having safe and accessible recreational parks around their community (4). While it is known that expansive park networks in neighborhood-built environments are positively associated with multiple aspects of health, well-being, and quality of life (4), populations' access and PA participation in such neighborhood parks is influenced by different variables such as individuals' ability, motivation to be physically active, and parks and leisure centers' proximity to their homes (5). For example, only adolescents living in neighborhoods with available recreational facilities have reported increased frequency and duration of performing PA (6). Insufficient numbers of parks and leisure centers have also been reported for US homes, with only one of five US homes having parks and leisure centers within half a mile radius (3). Nonetheless, reported association between increased PA levels and facility numbers within a neighborhood is inconclusive (7–11), showing either statistically positive (12), negative (7), or no significant association between the two variables (8, 9, 13). For example, Stewart et al., found no association between park's proximity and PA levels among an urban, mostly white, well-educated sample population that was well-served by neighborhood parks (9), though neither their socio-economic status nor their PA access was assessed. West et al., only focused on the largest American cities whilst reporting a positive correlation between park density and PA levels, which may not therefore be generalizable to smaller cities or rural areas (10). Whether and how PA participation is determined by facility access across US populations in large cities are largely unknown.

The objective of this study was to investigate whether access to free or low-cost recreational facilities was associated with meeting the AHA PA guidelines among US adults.

## Materials and Methods

### Study Design and Population

This was an analytical cross-sectional study utilizing demographics, built environment characteristics, and PA level data obtained from the Family Life, Activity, Sun, Health, and Eating (FLASHE) study conducted by the National Cancer Institute. The survey results in this publicly available online database encompassed a population of adult (*N* = 1,839) in the United States in 2017. A non-probability sample was recruited from all U.S. regions through the Ipsos Consumer Opinion Panel, a national market research firm. Based on sex, census division, household income and size, and race/ethnicity, eligible participants were balanced in the U.S. population. Within each household, one adolescent and one parent were randomly selected from eligible household members. Parents were considered eligible if there were at least 18 years of age and lived with at least one child aged between 12 and 17 years of age for >50% of the time. However, in this study we only included the parents. Via three surveys, these individuals were asked questions on demographics, health status, PA status, diet and built environment regarding both themselves and their adolescent (14). The main inclusion criteria included age of 18 years or above, participation in the 2017 FLASHE survey; and response to survey questions being studied including “My neighborhood has several FREE or LOW-COST recreation facilities, such as parks, walking trails, bike paths, recreation centers, playgrounds, etc.”, and one of the following: “How much time did you usually spend doing vigorous PA on one of those days?”, “How much time did you usually spend doing MODERATE PA on one of those days?” (15). Similarly, participants were excluded from the study if they either missed responses to the survey question assessing the independent variable described below, or if they did not have a response to any one of the two questions addressing the dependent variable.

### Variables

The main independent variable in this study was accessibility to free or low-cost recreational facilities. Perceived access to free or low-cost recreational facilities was assessed by the question “My neighborhood has several FREE or LOW-COST recreation facilities, such as parks, walking trails, bike paths, recreation centers, playgrounds, etc.” Participants could either respond “strongly agree,” “somewhat agree,” “somewhat disagree,” or “strongly disagree.” To create a dichotomous variable, the answer choices “strongly agree” and “somewhat agree” were combined into “agree”; and “strongly disagree” and “somewhat disagree” into “disagree.”

The main outcome variable was adherence to the AHA national PA guidelines. These guidelines recommend that adults should participate in more than 150 min of moderate intensity PA or more than 75 min of vigorous intensity PA per week (2). The self-reported quantitative weekly time spent doing walking, moderate or vigorous PA into one of two categorical variables: (1) at or above national guidelines or (2) below national guidelines. Quantitative data on vigorous activity were collected by the following questions: “During the LAST 7 DAYS, on how many days did you do VIGOROUS PA like heavy lifting, digging, aerobics, or fast bicycling?” and “How much time did you usually spend doing vigorous PA on one of those days?”. The amount of moderate PA was assessed by: “During the LAST 7 DAYS, on how many days did you do MODERATE PA like carrying light loads, bicycling at a regular pace, or doubles tennis? Do not include walking,” and “How much time did you usually spend doing MODERATE PA on one of those days?”, respectively. Individuals were considered above or equal to guidelines if they meet the guideline criteria for either vigorous or moderate PA. Those who do not meet guidelines for either of these two categories were categorized as below national PA guidelines.

This study included the covariates age, sex, level of education, overall health, BMI, ethnicity, employment status, hours of work per week, income, and number of years living at current residence. Age was assessed in the survey using the following categories: 18–34, 35–44, 45–59, and 60 + years-of-age (16). Sex was categorized into male or female. Levels of education were re-categorized into (i) high school or less (ii), some college but not a college degree, (iii) or a 4-year college degree or higher. Overall health status was re-categorized into excellent, very good, good, or fair/poor. BMI was calculated with height and weight and was categorized according to WHO criteria (17). A BMI <18.5 kg/m^2^ was considered underweight. A BMI between 18.5 and 24.99 kg/m^2^ was considered normal weight. A BMI between 25 and 29.99 kg/m^2^ was considered overweight. A BMI 30 kg/m^2^ and above was considered obese. Race/ethnicity was divided into four groups: Hispanic, Non-Hispanic Black, Non-Hispanic White, and others. Work status was categorized into not working, 0–30 h, 31–40 h, or 41+ h. Income was dichotomized into ≥$100,000 and < $100,000.

### Statistical Analysis

The data was analyzed utilizing Stata 16 software package (StatCorp LLC, College Station, Texas) (18). Primarily, a descriptive analysis was implemented to check the distribution of each variable. A chi-squared was used to perform a bivariate analysis of categorical data according to the main independent and the outcome variable to check for possible confounders. A collinearity check was then performed to check whether there is too much correlation between the variables. Lastly, unadjusted and adjusted logistic regression analyses were used to calculate the odds ratios and corresponding 95% confidence intervals. The Hosmer-Lemeshow test was used to assess the goodness-of-fit of the logistic regression models.

## Results

Table 1 presents the characteristics of the study participants of the 2017 FLASHE survey according to access to free or low-cost recreational facilities (*n* = 1,750). There was no statistically significant difference in the distribution of the age groups according to whether they had access to free or low-costs facilities or whether they did not (chi-square *p*-value 0.119). However, there was a statistically significantly higher proportion of men in those with access to free or low-cost facilities (30.6%) compared with those without access (19.4%; *p*-value < 0.001). In addition, participants' race/ethnicity, health status, BMI, education, work status and household income were statistically significantly different among those with access to free or low-cost recreational facilities compared with those without access (*p*-values < 0.05). For instance, those with access to recreational facilities had a higher proportion of being normal weight (39.3%) than those without access (31.5%). Moreover, those with access tend to have a higher perceived excellent or very good health compared with participants without access to free or low-cost recreational facilities (59.5 vs. 52.7%). Finally, 24% of the participants who had access to recreational facilities had a household income of at least 100,000 USD/year whereas the corresponding proportion was 15.1% in those without access (*p*-value < 0.001).

**Table 1:** Characteristics distribution of the 2017 FLASHE participants with and without access to free or low-cost recreational facilities.

**Characteristics**	**Access to free or low-cost facilities**				
	**Agree**		**Disagree**		***p*-value^a^**
	**(*n* = 1,079)**		**(*n* = 671)**		
	**%**	**(** ***n*** **)**	**%**	**(** ***n*** **)**	
**Age (years)**					0.119
18–34	10.9	(118)	11.3	(76)	
35–44	41.9	(452)	46.1	(309)	
45–59	44.7	(482)	39.2	(263)	
60+	2.5	(27)	3.4	(23)	
**Sex**					<0.001
Male	30.6	(330)	19.4	(130)	
Female	69.4	(749)	80.6	(540)	
**Race/ethnicity**					<0.001
Hispanic	8.3	(89)	5.3	(35)	
Non-Hispanic black	18.9	(202)	14.9	(99)	
Non-Hispanic white	67	(717)	73.6	(489)	
Other	5.8	(62)	6.2	(41)	
**Health status**					0.022
Excellent	17.4	(186)	13.9	(93)	
Very good	42.1	(450)	38.8	(260)	
Good	28.6	(306)	34.9	(234)	
Fair/poor	12	(128)	12.4	(83)	
**BMI**					<0.001
Underweight	1.4	(15)	1.5	(10)	
Normal	39.3	(419)	31.5	(209)	
Overweight	31.9	(340)	28.8	(191)	
Obese	27.4	(292)	38.2	(253)	
**Education**					<0.001
High school or less	16.5	(177)	20.8	(139)	
Some college	33	(355)	39.8	(266)	
A 4-year college degree	50.5	(543)	39.5	(264)	
**Work status**					<0.001
Not working	30.9	(332)	39.4	(263)	
0–30 h	15.6	(168)	15.3	(102)	
31–40 h	32.5	(349)	30.6	(204)	
41+ h	21	(225)	14.7	(98)	
**Household income**					<0.001
< $100,000	75.7	(807)	84.9	(564)	
≥$100,000	24.3	(259)	15.1	(100)	
**Time living**					0.561
0–3	21.91	(236)	20.45	(137)	
>3–10	36.68	(395)	39.1	(262)	
>10	41.41	(446)	40.45	(271)	

a *Chi-square test p-value*.

The unadjusted and adjusted associations between respondent characteristics and meeting AHA PA guidelines, which were stratified by access to free or low-cost recreational facilities are presented in Table 2. Before adjusting for covariates, participants who had access to free or low-cost recreational facilities had an odds ratio (OR) of meeting AHA PA guidelines of 1.63 (95% CI 1.33–1.99) when compared with participants who did not. After adjustment for the covariates, participants who had access to free or low-cost recreational facilities had statistically significantly higher odds of meeting the AHA PA guidelines compared with participants who disagreed with this statement [adjusted OR (aOR) 1.42; 95% CI 1.14–1.76]. The adjusted odds of meeting the AHA PA guidelines for the 35–44 age group was 0.77 (95% CI 0.54–1.11), for the age 45–59 age group 1.01 (95% CI 0.57–1.21) and for those ≥60 years-old 0.88 (95% CI 0.31–1.27) when compared with participants age 18–34 years-old. Participants who reported being male were 1.99 times more likely to meet the AHA PA guidelines compared with females (aOR 1.99; 95% CI 1.49–2.65). Being non-Hispanic white, Hispanic (aOR 1.31; 95% CI 0.83–2.07), non-Hispanic black (aOR 0.85; 95% CI 0.64–1.14) or other (aOR 0.8; 5 95% CI 0.54–1.35) was not associated with meeting AHA PA guidelines. Regarding health status, participants who reported excellent health status were 2.42 times more likely to meet the AHA PA guidelines (aOR 2.42; 95% CI 1.66–3.52), while those who reported very good health status were 1.47 times more likely to meet them (aOR 1.47; 95% CI 1.14–1.90) when compared with those with good health status. Furthermore, obese participants compared with those having a normal BMI had 39% reduced odds to meet PA guidelines (aOR: 0.61; 95% CI 0.46–0.80). Regarding education, having an educational level of high school or less, some college (aOR 1.09; 95% CI 0.81–1.47), or 4-year college degree (aOR 1.28; 95% CI 0.94–1.75) were all not associated with meeting the AHA PA guidelines. Household income status was not associated with increased or decreased likelihood of meeting the guidelines (aOR 1.32; 95% CI 0.98–1.79).

**Table 2:** Unadjusted and adjusted association between access to free or low-cost recreational facilities and meeting the American Heart Association's Physical Activity Guidelines for Americans, 2017 FLASHE survey.

**Characteristics**	**Meeting American Heart Association Physical Activity Guidelines**	
	**Unadjusted**	**Adjusted**
	**OR^a^ (95% CI^b^)**	**aOR^a^ (95% CI^b^)**
**Access to free or low-cost facilities**		
Disagree	Ref^c^	Ref^c^
Agree	1.63 (1.33–1.99)	1.42 (1.14–1.76)
**Sex**
Female	Ref^c^	Ref^c^
Male	2.23 (1.74–2.85)	1.99 (1.49–2.65)
**Health status**
Good	Ref^c^	Ref^c^
Excellent	2.89 (1.05–4.06)	2.42 (1.66–3.52)
Very good	1.68 (1.33–2.12)	1.47 (1.14–1.90)
Fair/poor	0.60 (0.44–0.83)	0.70 (0.50–0.99)
**BMI**
Normal	Ref^c^	Ref^c^
Underweight	0.63 (0.27–1.45)	0.59 (0.23–1.48)
Overweight	0.74 (0.58–0.96)	0.82 (0.62–1.08)
Obese	0.41 (0.32–0.53)	0.61 (0.46–0.80)

a *Age, Sex, Level of education, Overall health, BMI, Ethnicity, Hours of work per week, Household Income, Time living at current address*.

b *Odds ratio*.

c *Confidence interval*.

*^d^Reference group; p-value of the Hosmer-Lemeshow test for the adjusted model > 0.05*.

## Discussion

The main finding of the present study is that access to free or low-cost recreational facilities was positively associated with meeting the AHA PA guidelines in US adults. However, such association is also affected by sex, BMI, and health status, which were all associated with complying with the AHA PA guidelines. When controlling for covariates, males were twice likely to meet AHA PA guidelines compared with females, and participants with obesity were 39% less likely to meet AHA PA guidelines compared with those with normal BMI; while those with excellent health status were 2.42 times more likely to meet AHA PA guidelines when compared with those who reported a good health.

Our findings on subjective availability of outdoor recreational facilities and higher levels of total leisure-time PA are in line with previously reported associations. For example, in a survey in five European urban regions, Mackenbach et al. found a 25% difference in weekly min of total leisure-time PA between individuals with and without availability of outdoor recreational facilities. They postulated that the availability of these facilities seemed to be an important underlying mechanism for increased populations' PA, and the proximity was the main motivator for using recreational facilities (19). Several other reports have also showed that individuals who resided in neighborhoods with higher number of public parks and recreational facilities were more likely to be regularly physically active (7–11). One study described an increased amount of park-based PA in people with two parks within one mile (aOR 2.29) and three or more parks in one mile (OR 2.53) compared with those without any parks within one mile radius (8). West et al. reported that significant, positive correlations between park density and reported PA (*r* = 0.37, *p* < 0.01), and between park density and reported performing regular exercise (*r* = 0.35, *p* < 0.01) (10). While availability of public parks is now an established determinant in meeting PA guidelines, the scientific literature is inconsistent when analyzing an association between the proximity of public parks and PA levels. Kaczynski et al. reported that distance was not significantly associated with an increased likelihood of PA at the park (8, 13). Conversely, Cohen et al. reported that people living within one mile of the park averaged 38% more regular exercise sessions per week compared to those living further (IRR 1.38; 95% CI 1.04–1.84) and were four times as likely to visit the park once a week or more than those living further away (12). Therefore, reporting on the access to free and low-cost PA facilities and its positive association with meeting PA AHA guidelines in our study provides a new approach for increasing PA and associated long term health benefits in US populations.

Our study relied on a more subjective reporting when observing the association between access to free and low-cost recreational facilities and an increased likelihood of being physically active among US adults. An important distinction between the subjective (e.g., availability dependent on factors such as cost, safety, and hygiene) and objective availability (e.g., actual access or use) of recreational facilities has been previously reported with a significant association between the subjective availability of recreational facilities and leisure-time PA, however when the analysis was based on the objective availability (or reported use) of recreational facilities the association was not statistically significant (19). This may be due in part because individuals who report access to outdoor recreational facilities do so because they use them regularly—resulting in stronger associations with the perceived measure of PA levels (20).

The secondary outcomes of our analysis suggest that the healthier a person is the more likely they are to have met AHA PA guidelines (aOR = 2.42 and 1.47 for Excellent and Good Health, respectively), which was irrespective of the participants' age (Table 1). This may be explained by increased time spent exercising which has previously been associated with better health status (21). Therefore, increased time spent and awareness among those with perceived poor health is necessary when promoting PA access across US populations. Looking at BMI specifically, individuals with obesity were significantly less likely to meet the national PA guidelines which is contradictory to Lee et al.'s finding of obese individuals (BMI 25 and above) having no difference in level of PA when compared with individuals who are not obese (22). Our results most likely differ due to a difference in definition of obesity (over 25 BMI Asian cutoff point vs. over 30 BMI in US) but can also be explained by various reasons including lack of social support, lack of time, choosing a sedentary lifestyle, or lack of motivation to use PA facilities (23, 24). Studies that integrated similar BMI stratification such as Hemmingsson et al. found similar results to ours of a clear association between BMI and level of PA (25). Sex had the largest impact on the odds of meeting PA guidelines where men were twice at odds of meeting PA guidelines than women. This finding is in line with previous studies that describe increased PA in males compared to females (26). It has been proposed in past studies that societal influences are stronger on males than females regarding PA which can explain the increased likelihood of meeting guidelines for men (27).

Naturally, our study has some limitations. First, our cross-sectional study design lacks the ability to implement causality. While we can illustrate an association between two variables, we are unable to correlate the two due in part to the possibility of reverse causality. The study design also introduces the possibility of various types of bias. Our study measured PA by a self-reported questionnaire, which may over- or underestimate true PA behavior. Thus, there is potential for social acceptability biases. This may be illustrated in part by the larger than expected percentage of individuals that met requirements and the few impossible and few possible but improbable outcomes reported. Nevertheless, the PA questions in this study were validated against an objective PA accelerometer data (*r* = 0.52, *p* < 0.01) (28). Similarly, while this study makes an important distinction from past studies in that it reports people's access to free or low-cost facilities, we recognize that it is somewhat vague in its definition of accessibility. While it is important to recognize that there is an association between access and PA, there is no concrete quantitative data to be used by local governments to best implement new policies. A strength of this study was that it included a national sample from a large, regionally diverse sample of the U.S. population, although not nationally representative. The national sample allowed taking into consideration variation in environmental contexts, socio-demographics, and behaviors. For example, access to recreational facilities vary between most U.S. states. Therefore, using a national sample allows for more generalizable results than a study located in a specific city or state.

In conclusion, our data provides various recommendations that can be extrapolated for use by health professionals, public health officials and the public. We recommend increasing the availability of facilities such as parks, trails, fields, and courts in various neighborhoods, and to increase awareness of the availability of these facilities, perhaps *via* targeted social media, newspapers, magazines, and local television networks so that PA is promoted across wider populations especially females and those with poorer health conditions including those with obesity. Future studies could focus on improving access to free or low-cost recreational PA facilities by understanding individuals' barriers and facilitators to using such facilities and using more objective PA assessment approaches.

## Data Availability Statement

Publicly available datasets were analyzed in this study. This data can be found here: https://cancercontrol.cancer.gov/brp/hbrb/flashe-study.

## Author Contributions

LA, RG, MM, AA, and NB contributed to the conception and design of the study. GC and PR supervised data collection. LA, RG, and MM organized the dataset and wrote the first draft of the manuscript. LA, RG, MM, GC, and PR performed the statistical analysis. AA, GC, PR, and NB revised the manuscript. All authors contributed to manuscript revision, read and approved the submitted version.

## Conflict of Interest

The authors declare that the research was conducted in the absence of any commercial or financial relationships that could be construed as a potential conflict of interest.

## Publisher's Note

All claims expressed in this article are solely those of the authors and do not necessarily represent those of their affiliated organizations, or those of the publisher, the editors and the reviewers. Any product that may be evaluated in this article, or claim that may be made by its manufacturer, is not guaranteed or endorsed by the publisher.

## Abbreviations

AHA: American Heart Association
BMI: Body Mass Index
CI: Confidence Interval
FLASHE: Family Life, Activity, Sun, Health, and Eating
HHS: U.S. Department of Health and Human Services
OR: Odds Ratio
PA: Physical Activity
Ref: Reference Group
Rs: Spearman's Rank Correlation Coefficient
US: United States
WHO: World Health Organization.
